# Mechanisms for DNA Interplay in Eukaryotic Transcription Factors

**DOI:** 10.1146/annurev-biophys-071524-111008

**Published:** 2025-01-29

**Authors:** Victor Muñoz, Rama Reddy Goluguri, Catherine Ghosh, Benjamin Tanielian, Mourad Sadqi

**Affiliations:** 1 CREST Center for Cellular and Biomolecular Machines, University of California, Merced, California, USA; 2 Department of Bioengineering, University of California, Merced, California, USA; 3 Department of Biochemistry, Stanford University, Palo Alto, California, USA; 4 Chemistry and Biochemistry Graduate Program, University of California, Merced, California, USA

**Keywords:** eukaryotic transcription factors, promiscuous recognition, transcription antenna, DNA scanning, conformational control, nucleosome targeting

## Abstract

Like their prokaryotic counterparts, eukaryotic transcription factors must recognize specific DNA sites, search for them efficiently, and bind to them to help recruit or block the transcription machinery. For eukaryotic factors, however, the genetic signals are extremely complex and scattered over vast, multichromosome genomes, while the DNA interplay occurs in a varying landscape defined by chromatin remodeling events and epigenetic modifications. Eukaryotic factors are rich in intrinsically disordered regions and are also distinct in their recognition of short DNA motifs and utilization of open DNA interaction interfaces as ways to gain access to DNA on nucleosomes. Recent findings are revealing the profound, unforeseen implications of such characteristics for the mechanisms of DNA interplay. In this review we discuss these implications and how they are shaping the eukaryotic transcription control paradigm into one of promiscuous signal recognition, highly dynamic interactions, heterogeneous DNA scanning, and multiprong conformational control.

## INTRODUCTION

Transcription factors control gene expression by binding to certain DNA sites on the regulatory elements of target genes in order to recruit or block the transcriptional machinery. This function requires meeting important mechanistic challenges in terms of sequence recognition, target site occupancy and residence time, genomic search efficiency, and quality control.

Sequence recognition is generally considered a binary process in which the factor can select its cognate motif among all other possible DNA sequences. The foundations for recognition are specific interactions made between amino acid side chains in the factor and nitrogenous bases of the cognate motif (47), including DNA shape contributions (109). The kinetics of the interactions with the target DNA are important because the dissociation rate determines the duration of the action, and the target association rate ultimately controls how nimble the gene expression response is. The latter comes with important search implications since transcription factors must scan large fractions of the genome to locate their target sites. Finding these targets quickly and consistently requires specialized scanning mechanisms. In general terms, this search occurs via a facilitated diffusion process that combines conventional 3D diffusion-collision kinetics with a 1D diffusive motion along the DNA that reduces the search dimensionality (126). Facilitated diffusion has been studied in depth, with the use of single-molecule tracking experiments in vitro (53) and in vivo (34), theory (69), computer simulations (11), nuclear magnetic resonance (60), and biochemical experiments (39, 59). The search-recognition process can be further optimized by coupling conformational changes in the factor with the energetic environment provided by the DNA (8, 121). This control mechanism has been studied at length with molecular simulations (68, 74, 117).

Such molecular elements provide a reasonably complete understanding of the functioning of prokaryotic transcription factors, which bind specifically to short operator elements located near the promoter of an operon to control the concerted transcription of all the genes within the operon (36). In this review we discuss the basic mechanisms for DNA interplay used by eukaryotic transcription factors (ETFs), focusing on their specific functional needs and environmental constraints. Although the same elements discussed above apply here, the underlying mechanisms need to be reconsidered due to the fundamental differences of gene expression in eukaryotes.

For instance, ETFs operate amid chromatin remodeling events (16) further regulated by DNA methylation (113) and histone post-translational modifications (93). Nucleosomes are the basic chromatin units composed of an octamer of four histones (H2A, H2B, H3, H4) that wrap approximately 147 bp of DNA in two gyres, forming a toroid (25). The positioning of the nucleosomes determines the accessibility to DNA in the eukaryotic cell (105). Therefore, the impact of chromatin dynamics on ETF function cannot be ignored.

Another key difference lies in the genetic regulatory elements. In eukaryotes, regulatory elements are actually long DNA regions difficult to define solely by sequence analysis (90). These regions can be localized to a nearby gene (*cis*, intergenic regions) or longer distances along or between chromosome territories (*trans*, enhancers) (133). Whether *cis* or *trans*, eukaryotic regulatory regions contain cognate sequence motifs for many ETFs (106), as well as clusters of imperfect motifs for ETFs with master regulator roles (28, 75, 135). Such clusters of imperfect motifs, or short tandem repeats (STRs), constitute 5% of the regulatory regions in humans (103), being particularly abundant in the noncoding regions of genes (43). STR-rich regions are clearly important for gene regulation since they exhibit increased multi-ETF occupancy in vivo (82, 83). Furthermore, removing STR clusters from enhancers destabilizes cell fates during embryonic development (24, 40), whereas mutations in STRs have been implicated in several diseases (56, 94).

Finally, ETFs are largely composed of intrinsically disordered regions (IDRs) (85, 124, 134). The IDRs introduce new transcriptional control functionalities by facilitating flexible, multivariate interactions with DNA (13) and other cofactors (115). The highly dynamic interactions mediated by IDRs can induce liquid-liquid phase separation, leading to biomolecular condensates containing multiple ETFs and other partners organized as transcription hubs (85, 115).

An emerging theme from recent results is that these operational characteristics have profound, largely unforeseen, implications for the mechanisms of DNA interplay used by ETFs. In the following sections we discuss these results and how they are reshaping our understanding of ETF function away from the classical prokaryotic-centric viewpoint.

## A PROMISCUOUS DNA RECOGNITION CODE

**Figure 1:**
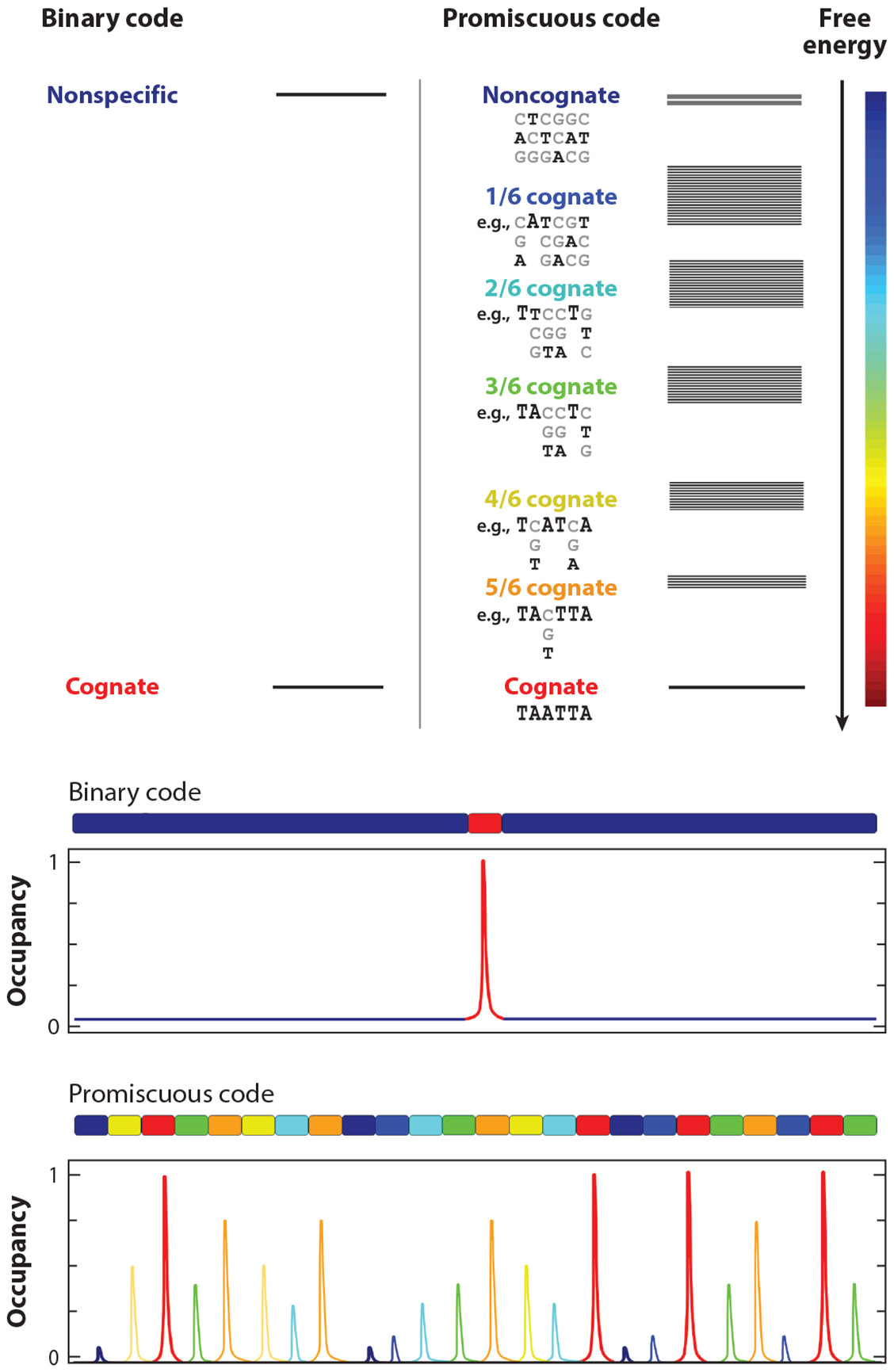
Differences between a binary and a promiscuous DNA recognition code. The classical binary code discriminates between a given DNA sequence motif and all other sequences. The binding free energy spectrum consists of two bands, one band of high affinity for the cognate motif and another band of much lower affinity for any other sequence. This code results in a single occupancy peak at the target site. A promiscuous recognition code produces a spectrum with different affinity tiers, each corresponding to a certain number of base deviations from the cognate motif. Each tier contains multiple bands accounting for specific differences in location and base substitution. For example, noncognate A-to-T and T-to-A changes result in a much smaller free energy perturbation for enHD than do changes to G or C, suggesting that its recognition code has important contributions from the shape of the minor groove. A promiscuous recognition code results in a distributed pattern, with many local DNA sites exhibiting significant occupancy levels as shown in the bottom panel. In such a case, the elimination of one high-affinity site does not produce large changes in overall occupancy because the sites at the flanking regions buffer the effect. Abbreviation: enHD, the DNA binding domain of the eukaryotic transcription factor Engrailed. Figure adapted from images created with BioRender.com.

The sequence logo for a given transcription factor is usually determined by in vitro high-throughput binding-selection methods, like protein binding microarrays (PBMs) (89), systematic evolution of ligands by exponential enrichment (SELEX) (61), and mechanically induced trapping of molecular interactions (MITOMIs) (42, 81). These assays use libraries of oligonucleotide sequences to determine the probability of finding each of the four bases at each position along the pool of bound oligonucleotides. The resulting data are tabulated as a position weight matrix (PWM). Significantly, the PWMs determined for a vast number of transcription factors indicate well-defined sequence logos (4). This is true for prokaryotic transcription factors with 12–30-bp-long sequence logos (33), as well as for ETFs, which feature much shorter cognate motifs of 6 to 10 bp (6). The commonality in defined logos has buttressed the idea that DNA recognition is binary, where the factor binds to its cognate motif with high affinity and to all other possible DNA sequences with a uniform, much weaker affinity (Figure 1).

The in vivo DNA binding preferences of ETFs are usually examined via chromatin immuno-precipitation followed by sequencing (ChIP-seq) experiments (44, 66). The puzzle has been that ChIP-seq in vivo occupancies of ETFs correlate poorly with the expectations from their in vitro PWMs (27, 58, 131). Recent efforts have used machine learning approaches to improve the predictions of in vivo ETF occupancy data (3, 4, 127). But an important factor to consider is whether the in vitro profiling methods, which are essentially competition assays, may introduce excessive bias toward the highest-affinity binding sequences. We looked into this possibility by analyzing the equilibrium binding thermodynamics of enHD, the DNA binding domain (DBD) of the ETF Engrailed, to its cognate motif TAATTA and variations that were embedded in relatively long DNA sequences (75–300 bp) (17). The effects caused by cognate site mutations did follow the PWM preferences, but the overall affinity changes were many orders of magnitude smaller. For instance, a highly noncognate site that was expected to produce a 20 billion drop in affinity according to the PWM reduced binding by only a factor of 40 (17). Global analysis of the multivariate binding data with a structure-based statistical mechanical model revealed that this behavior reflects a recognition code that is highly promiscuous. The code for Engrailed is such that each single-base deviation from the cognate motif produces a slight cumulative drop in affinity that depends on both position and type of substitution, resulting in a broad, multitiered-binding free energy spectrum (Figure 1). As a consequence, the effects on overall occupancy of the flanking DNA are large and buffer the drops in affinity caused by cognate site mutations. Importantly, the in vitro–derived statistical mechanical model correctly identified all the ChIP-seq *Drosophila*’s genome fragments occupied in vivo by Engrailed as having high global affinity, although most of them lacked cognate motifs (17). A key implication is that promiscuous recognition can help explain the complex in vivo occupancy patterns found for ETFs.

From a structural standpoint, a promiscuous recognition code conforms with the use of a 6-bp short binding site, which does not provide many opportunities for the protein to form a network of base-specific interactions. This structural argument suggests that promiscuous recognition is likely to be widespread in ETFs given their generally short cognate motifs (6). For Reb-1, for example, the removal of the specific site on a 147-bp DNA dropped the affinity by only 10-fold (31), consistent with the buffering of occupancy by binding to promiscuous sites. Moreover, the recent incorporation into in vitro high-throughput selection methods of strategies for reducing the bias toward the highest-affinity binding is providing additional evidence of promiscuity in ETF recognition, whether by detecting submaximal binding (9), exploring the effect of flanking short tandem repeats (57), or more widely applying new tools designed to detect a broad range of midlevel affinities (110). Significantly, the binding spectra that have been obtained for ETFs other than Engrailed with the last approach are consistently broad, in agreement with the promiscuous recognition scenario depicted in Figure 1. There is indeed a growing realization that a broad spectrum of binding affinities provides important functional advantages in the context of eukaryotic transcription. For instance, it has been argued that promiscuous recognition helps select between paralogous ETFs that share sequence logos but have distinct biological functions through their different preferences for low-affinity sites (71, 116).

## TRANSCRIPTION ANTENNAS FOR GENE TRACKING AND COLOCALIZATION

As discussed in the Introduction, the STR clusters that fill eukaryotic regulatory regions play important functional roles in the control of gene expression. However, the molecular mechanisms behind their operation have been largely mysterious (43). Several STR effects have been proposed from theoretical considerations, including symmetry-enhanced binding (1, 114) and target search accelerations (73, 119). On the other hand, the realization that ETFs can recognize DNA promiscuously offered an immediate mechanism for gene tracking or colocalization. On the basis of the thermodynamic characterization of promiscuous binding in enHD, we argued that the STR clustered architecture of eukaryotic regulatory regions will necessarily result in myriad mid-affinity binding events that enhance overall ETF occupancy by spreading it out over many local sites rather than populating just one site (Figure 1). These mid-affinity binding events can act as a dynamic attractor that colocalizes ETF molecules with the genomic regions containing the relevant genes and/or regulatory elements. We termed this type of mechanism a transcription antenna (17).

A gene-tracking mechanism like transcription antennas is essential for eukaryotes, since ETFs typically control multiple genes, sometimes hundreds of them, that are spread around the genome and often in different chromosome territories (133). Likewise, each gene is controlled by a group of ETF partners (106). Because ETFs are present in tens of thousands of copies per cell (10), transcription antennas can serve to colocalize a defined number of ETF molecules of each kind with every genomic region of interest, making them available on demand (17). Binding to STRs also has been recently reported for ETFs Pho4 and MAX, where the addition of STRs flanking a cognate site increased DNA occupancy by mostly enhancing the global association rate (57). These results indicate that Pho4 and MAX can also exploit STRs to generate antenna effects. Modulation of occupancy by the sequence flanking the cognate site was noted in a previous single-molecule force spectroscopy study of Egr1, an ETF containing three zinc finger domains (111). While more local and subtle, the reported context-dependent modulation of Egr1 binding is also consistent with antenna effects driven by promiscuous recognition.

## DNA INTERACTION KINETICS

**Figure 2:**
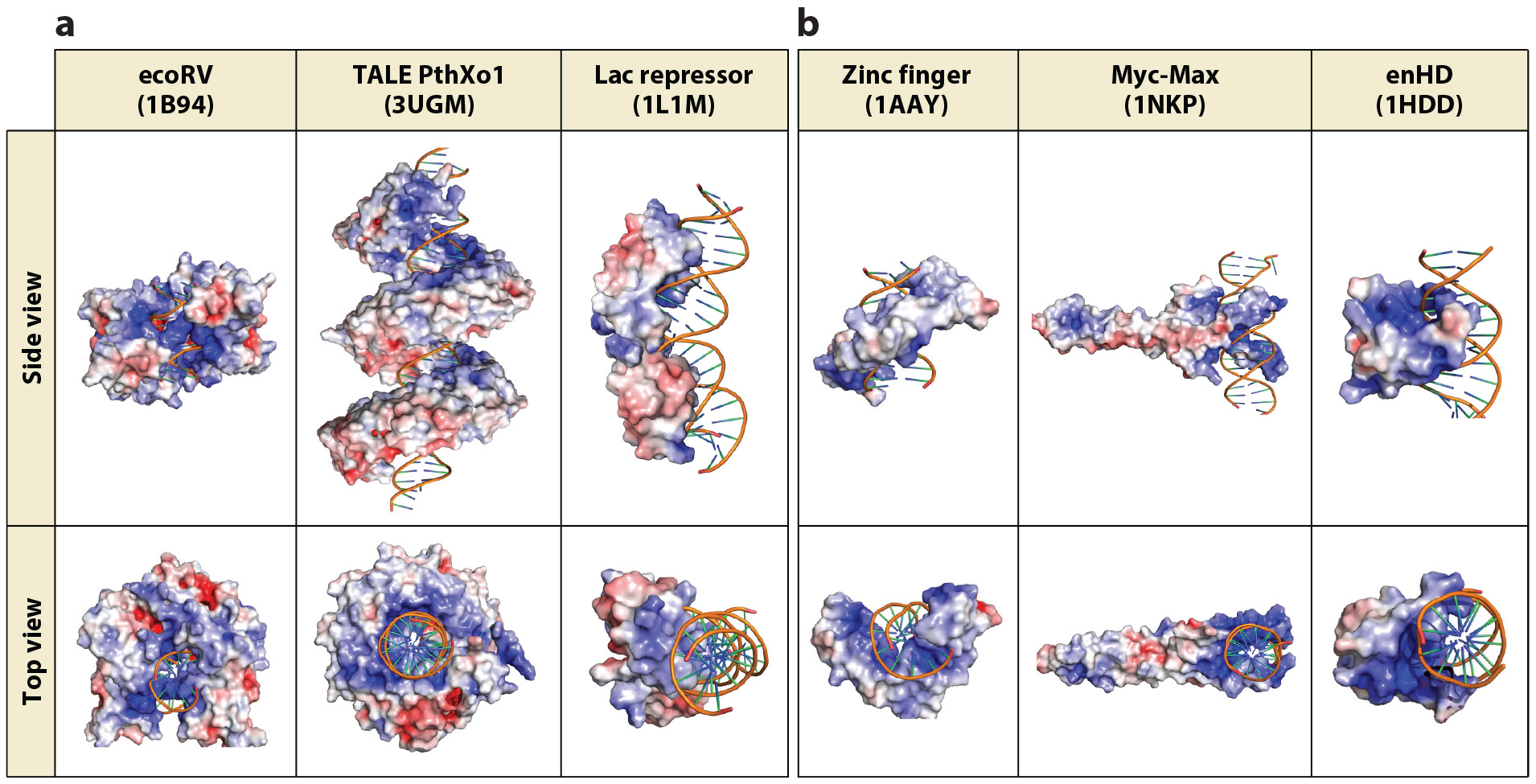
DNA interaction interfaces of several DNA binding proteins. Top and side views for each complex are shown, with the protein structure displayed in an electrostatic surface representation and the DNA displayed as a ribbon. The Protein Data Bank codes for the structures are provided in parentheses. Panel *a* shows examples of a restriction enzyme, a genome-editing enzyme, and the prokaryotic Lac repressor dimer. Panel *b* shows examples of eukaryotic DNA binding proteins, including a zinc finger, a basic zipper, and a homeodomain. Abbreviation: enHD, the DNA binding domain of the eukaryotic transcription factor Engrailed. Figure adapted from images created with BioRender.com.

The rates for DNA association (*k*_on_) and dissociation (*k*_off_) are determined to a large extent by the interaction interface between the protein and the cognate DNA. These interfaces are quite variable for different DNA binding proteins, with some examples shown in Figure 2. In principle, the *k*_off_ should be inversely proportional to the number and strength of the DNA interactions that break upon dissociation. To a first approximation, this would indicate that the smaller the interaction surface is the faster the dissociation will be. This relationship is significant because ETFs feature smaller contact interfaces with DNA (Figure 2b). There is indeed some evidence supporting such a relationship. The prokaryotic LexA, Lac, and λ repressors and the yeast Gal4 recognize sequence logos of 24 to 18 bp, and their *k*_off_ are all <10^−3^ s^−1^ (80, 107, 129). These factors reside on their target sites for tens of minutes. In contrast, most ETFs have much shorter resident times, like Egr1, which has a 9-bp logo and *k*_off_ = 0.05 s^−1^ or 20 s residence time (65), and Rap1 and Reb1, which have 7–8-bp logos and k_off_ close to 1 s^−1^ (31, 95). Furthermore, in recent experiments we found that enHD with a 6-bp logo dissociates from its cognate site in just milliseconds (C. Ghosh, M. Sadqi, Z. Wang & V. Muñoz, manuscript in preparation). The implication is that ETFs use a much more dynamic DNA interplay than do prokaryotic transcription factors. The faster dynamics help explain the results from single-molecule ETF tracking in live cells, which show that ETFs stay steady/bound for about 1 s on average (7, 50, 98). The in vivo dwell-time distributions have extended tails, with subpopulations of much longer-lived events than were initially identified with cognate site binding (46). However, similarly extended time distributions have been reported for prokaryotic factors ectopically expressed in mammalian cells, where they lack functional sites (102). Therefore, the most likely interpretation for the longer-lived binding events is that they represent slow dissociation not from one high-affinity site but from multiple local recaptures (46), consistent with the ETF being colocalized with a transcription antenna.

Since *K*_D_ = *k*_off_/*k*_on_, ETFs must trade the inherently faster dissociation rates with drops in affinity or compensate them with accelerated association rates. The prokaryotic Lac repressor binds its cognate site with 10 pM affinity (49) and *k*_on_ = 7·10^9^ M^−1^s^−1^ (107). Both the affinity for cognate DNA and the concentration in living cells of ETFs are typically near 1 nM (10). This affinity value implies an overall 100-fold affinity trade-off relative to the Lac repressor, leading to expected *k*_off_ values of approximately 0.05 s^−1^. For ETFs that dissociate with rates significantly higher than 0.05 s^−1^, it follows that the *k*_on_ should also be faster than the Lac repressor reference. What factors could make the 3D diffusion-collision rate faster? The most obvious options are the degree of enclosure of the DNA interface, and the strength of the electrostatic attraction. A more open interface should increase the probability of reactive collisions, whereas a strongly positive protein can accelerate its diffusion-collision limit by electrostatic steering (54). In this regard, we note that ETFs use more open DNA interfaces and tend to have a higher positive charge density at the interface (Figure 2). Both factors point to potentially faster association rates. The difficulty lies in extricating the 3D diffusion-collision rate from other processes involved in facilitated diffusion, particularly 1D scanning and intersegment transfer (discussed below). These combined factors give rise to an apparent second-order target association rate defined as *k_a_* = *S*ρη*k*_on,*N*_ (59). In this equation *S* is the average distance between the target and any other DNA site from which the factor can slide without dissociating; ρ is the fraction of factor molecules not trapped in sites that are too far away to reach the target through sliding; η is the acceleration due to intersegment transfer; and *k*_on,*N*_ is the association rate constant for binding to any site (59). This treatment was applied to Egr1 using kinetic binding data for DNA molecules of different lengths containing one cognate site at varying ionic strengths (37, 38, 112, 137). The *k*_on_ so obtained for Egr1 is approximately 10^7^ M^−1^s^−1^ (137), which is actually much slower than the Lac repressor reference. This comparison immediately points to the reported Lac repressor *k*_on_, which was measured on long DNA, having indeed large contributions from 1D diffusion (126).

## MECHANISMS FOR SCANNING NAKED DNA

**Figure 3:**
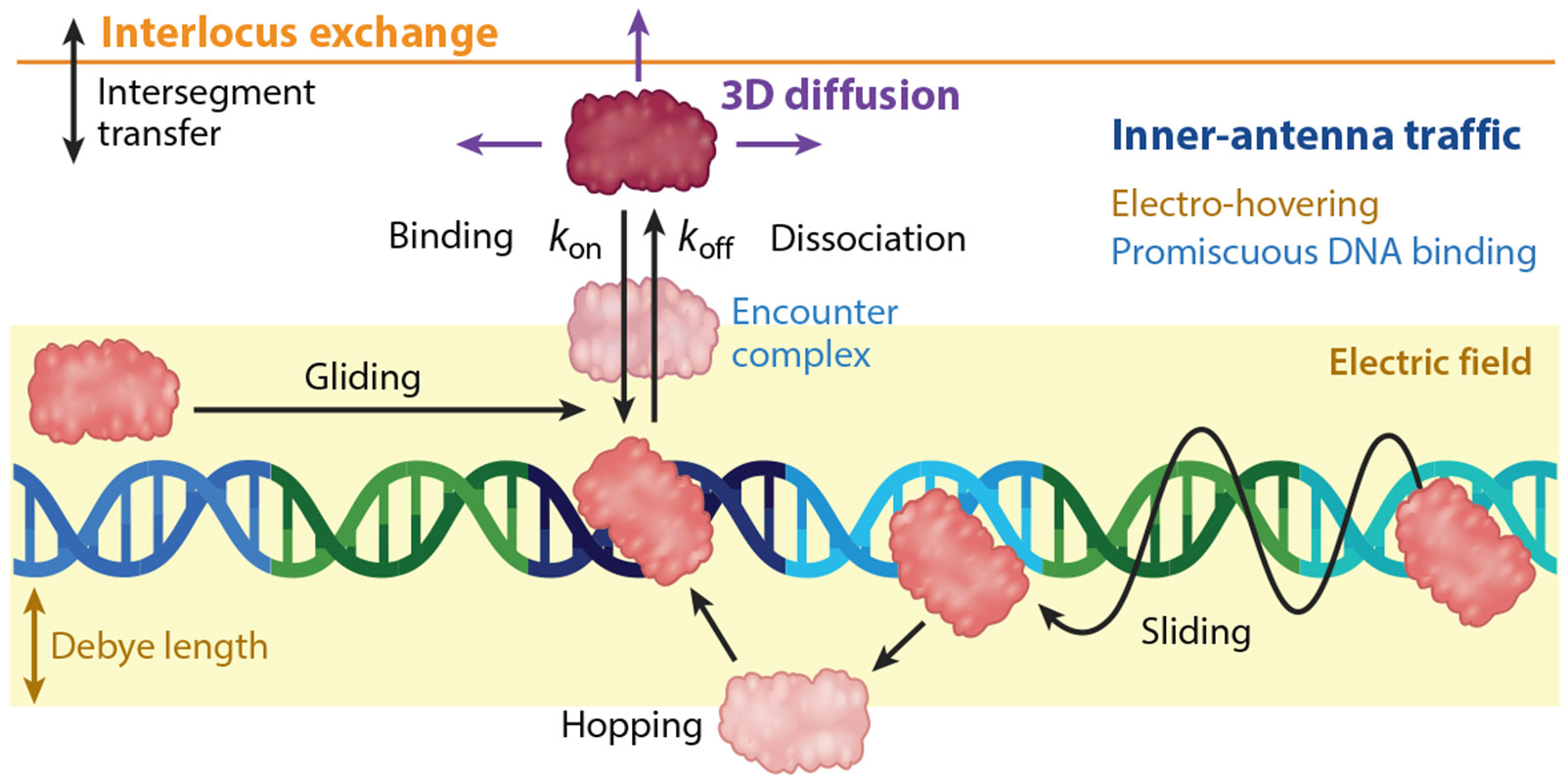
Proposed model for naked DNA scanning by ETFs that use a promiscuous recognition code and an open DNA interface. The different colored sites on the DNA represent the varying partial cognate-like affinities of the STR clusters present in a transcription antenna. The yellow-shaded region represents the reach of the DNA electric field that causes electro-hovering for a sustained 1D scanning motion on the DNA without mechanical clamping. The orange line represents the limits of the transcription antenna dynamic attractor. ETF molecules can escape from this attractor via classical 3D diffusion or via intersegment transfer, enabling the exchange of ETF molecules with other gene loci. Abbreviations: ETF, eukaryotic transcription factor; STR, short tandem repeat. Figure adapted from images created with BioRender.com.

DNA scanning has been amply studied on the Lac repressor and several enzymes, but much less so from the viewpoint of ETFs in the context of active genomic DNA, that is, naked rather than packed into chromatin. On average, approximately 2.5% of the 3 Gbp human genome is transcriptionally accessible (99). The transcriptionally accessible regions are dynamically controlled by chromatin remodeling (16) and epigenetic factors (62). Therefore, ETFs face a scanning landscape of approximately 75 Mbp of DNA containing *cis*-regulatory regions, active genes, and *trans*-enhancers dynamically distributed among chromosome territories. Figure 3 summarizes a proposed scenario for ETF scanning of active DNA that considers recent findings. The first distinction from the conventional prokaryotic search scenario comes from the promiscuous recognition code of ETFs and the STR clusters of eukaryotic genomes. Under such conditions, most ETF scanning traffic likely occurs within transcription antennas (regions containing STR clusters), whereas 3D diffusion and intersegment transfer provide ways for transferring to other gene loci. Inside the antenna, we anticipate ETFs alternating between 3D diffusion-collision and 1D diffusion composed of three microscopic scanning modes: rotational sliding (12, 126), hopping (70, 86), and gliding (21) (see Figure 3). For ETFs we also expect a major role for the DNA electrostatic field in sustaining 1D diffusion (39). This is so because the small and open DNA interfaces of ETFs (Figure 2b) tend not to encircle the DNA and thus cannot mechanically support sliding. In addition, promiscuous recognition should make the binding landscapes of regulatory regions rugged and thus difficult to slide on.

p53 was the first, and for a long time only, ETF looked at in terms of DNA scanning (122, 123). Wang et al. (129, 130) observed a scanning behavior similar to that of the prokaryotic Lac repressor. Namely, both factors scan DNA for seconds at a time with diffusion coefficients (*D*) that are relatively close to the sliding speed limit. These properties indicate little scanning friction, as expected for a binary search-recognition code (Figure 1). But we note that p53 is a tetramer that recognizes a long sequence motif split into two 10-bp halves separated by up to 13 bp (132) that is long enough to elicit specific binding. p53 also uses separate domains for recognition and nonspecific scanning, where the recognition domain alone proved incapable of sliding (122). Furthermore, the p53 tetramer fully encloses the DNA (67), offering an excellent clamping interface for sliding. In other words, p53 appears as a special case in terms of DNA scanning by ETFs and is significantly different from the scheme detailed in Figure 3.

The question then was, How do ETFs that recognize short sequence motifs scan DNA? We addressed this question using enHD as a paradigm of promiscuous recognition and the open DNA interface. We found that this ETF performs extensive 1D diffusion on DNA at speeds comparable to those of p53 (51, 52). Our results indicated that enHD scanning is sustained by a long-range attraction to the DNA electric field, which substitutes for mechanical clamping (51, 52). The scanning speed of enHD was a surprise because the λ DNA we used as a scanning substrate presents an extremely rugged binding landscape for Engrailed, owing to promiscuous recognition. In fact, we estimated that enHD scans DNA approximately 200,000-fold faster than expected for a continuous slide in such a binding landscape. We also discovered that enHD alternates stochastically between a 100 ms scanning phase and a 10 ms redeployment phase consisting of long jumps of 530 bp along the DNA. During the scanning phase enHD covers 675 bp using a heterogeneous motion that likely combines hops, slides, and glides, but that is too transient and localized to be resolved with current single-molecule tracking methods. This hybrid mechanism is appealing because it appears perfectly suited for scanning transcription antennas without getting trapped into local STR clusters. The long jumps provide an escape mechanism to move from cluster to cluster within regulatory loci of interest, which are in fact organized in the genome as islands within archipelagos (97), or to facilitate interlocus exchange.

## PROTEIN CONFORMATIONAL TRANSITIONS AND DNA RESHAPING

**Figure 4:**
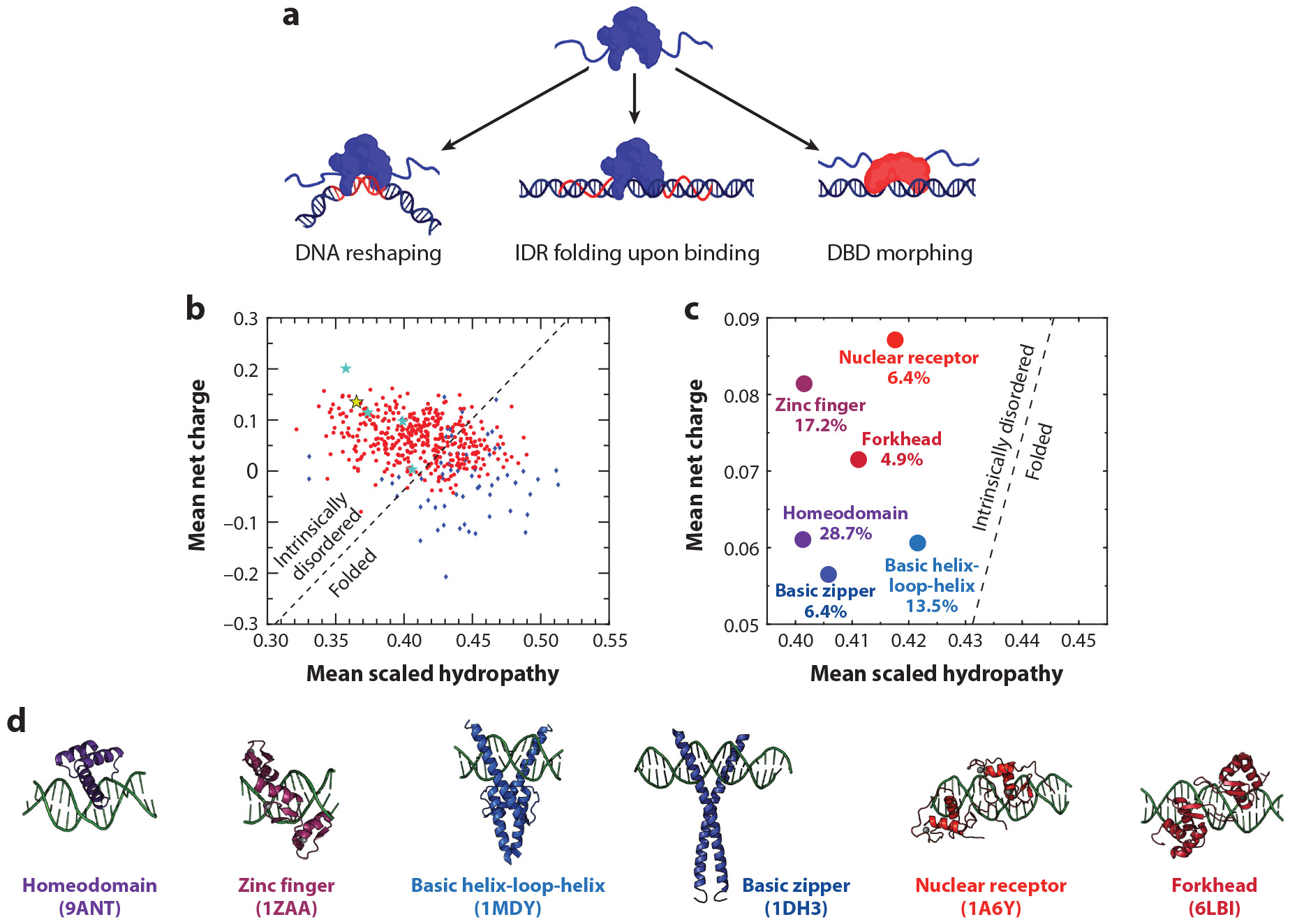
Protein conformational changes and DNA reshaping. (*a*) ETF binding to cognate DNA can alter the local DNA shape, trigger folding on the DNA of IDRs flanking the DBD, or induce a conformational change in the DBD. (*b*) Mean scaled hydropathy plotted against mean net charge for a subset of single-domain proteins from an experimental folding database (84) (*blue diamonds*) and for a database of eukaryotic DBDs tested for nucleosome targeting in a high-throughput survey (140) (*red circles*). The black dotted line represents the boundary between folded and intrinsically disordered proteins. The stars represent proteins from the folding database that are in fact DBDs, with enHD shown in yellow and the others in cyan. (*c*) Centroids for the six largest eukaryotic DBD families from the Zhu et al. (140) database. (*d*) DNA-bound structures of examples of each DBD family. Abbreviations: DBD, DNA binding domain; enHD, the DNA binding domain of the eukaryotic transcription factor Engrailed; ETF, eukaryotic transcription factor; IDR, intrinsically disordered region. Figure adapted from images created with BioRender.com.

ETF binding to DNA can result in conformational transitions of the ETF and/or in DNA reshaping (Figure 4a). ETF binding is often associated with sequence patterns that cause DNA distortions, such as reduced DNA duplex stability, breathing of the flanking regions, or a narrow minor groove (109, 136). Consideration of DNA shape does indeed improve the prediction of ETF binding patterns in vivo (78, 87, 139). Furthermore, a high-throughput study demonstrated that ETFs bind more strongly in vivo to DNAs that were modified to include mismatched base pairs that promote DNA structural distortions resembling those naturally induced by ETF binding (2). These results confirmed that DNA reshaping plays a major role in ETF target discrimination. It has also been noted computationally that DNA reshaping could in turn make the ETF skip its target multiple times, slowing down recognition (108). ETF binding can also bend the DNA at the cognate site, as was shown for p53 (35, 104). Large mechanical effects such as these require a large surplus of binding free energy and a mechanical lever. Hence, they might be feasible only for multimeric ETFs with a long recognition site, like p53, or via the collective action of several ETF molecules binding together locally.

The DNA environment can also trigger conformational changes in the factor, which can be used accordingly to control the DNA scanning process by switching between search and recognition modes (121) (Figure 4a). In ETFs, such conformational changes are typically connected with their abundant IDRs (85, 134). The idea is that IDRs flanking the DBD can expand the search radius by binding nonspecifically at a distance (76), thus enabling a swinging search motion (128). p53 has two IDRs flanking the central DNA recognition core. The positively charged C-terminal IDR appears to play such a role (64, 122), whereas the negatively charged N-terminal IDR interacts transiently with the core domain as a way to increase the core’s recognition specificity (72). Other examples are Msn2 and Yap1, whose long IDRs are required for colocalization in vivo with most of their known target promoters (14).

The DBDs of many ETFs have defined structures that do not seem to change in the X-ray crystal structures of their complexes with cognate DNA (47). In this context, the DBD could still exert control by modulating its conformational dynamics. This phenomenon was first identified in the Lac repressor, with a rigid binding interface for cognate and a flexible interface for nonspecific DNA (63), where the flexible interface enhances scanning in simulations (79). There are similar examples for ETFs, such as NF-κB, which requires conformational heterogeneity that is arrested by IκBα to bind DNA (19), and the Myc-associated transcription factor (MAX) (120). An alternative mechanism postulates that the DNA electrostatic field can produce a tidal force that partially disorders an otherwise folded DBD when in proximity, thereby facilitating DNA scanning (76) (Figure 4a). Using molecular simulations, we found evidence for this phenomenon in enHD (21). We then discovered experimentally that enHD experiences a large conformational change when it binds cognate DNA at physiological conditions, whereas higher ionic strengths block it (26). We found more recently that enHD also responds to noncognate DNA by changing to a conformation that is different from the conformation it adopts when induced by cognate DNA (B. Tanielian, M. Sadqi & V. Muñoz, manuscript in preparation). Here it is important to note that whereas enHD is well-structured (22), its native stability is marginal (88). Furthermore, enHD features a net charge and hydropathy index of an intrinsically disordered protein (125) (Figure 4b), which helps explain its morphing capabilities in response to DNA. In this regard we find it remarkable that most of the known eukaryotic DBDs fall on the intrinsically disordered side of the net charge versus hydropathy scale (Figure 4b). The trend is true, on average, for each of the DBD families, including homeodomains, zinc fingers, nuclear receptors, forkheads, basic helix-loop-helix domains, and basic zippers (Figure 4c). Part of the reason is the positive net charge that the DBD uses to interact with DNA, but these domains have characteristically low hydropathies as well. The combination of both traits suggests that eukaryotic DBDs may have been specifically selected through evolution to be conformationally responsive to the negative electric field of the DNA.

## FUNCTIONING AROUND CHROMATIN: AVOIDING, TARGETING, AND ACTING ON NUCLEOSOMES

In the cell, nucleosome positioning determines the accessibility of DNA sites for ETF binding (105). Displacing nucleosomes does in fact require significant work. For instance, under stretching force, nucleosome DNA unwraps from the histone core in two steps: an uncooperative low force (~4 pN) transition that results in a 60–65-bp extension, attributed to pulling the entry/exit DNA region away from the nucleosome core, and a cooperative high force (20–35 pN) transition that results in a 75–80-bp extension that fully releases the remaining toroidal wrapping (18, 29, 55, 92, 100, 118). In vivo, nucleosome (dis)assembly is an energy consumption process carried out by chromatin-remodeling enzymes, some of which have been looked at with single-molecule force techniques (18, 118). But nucleosomes are also highly dynamic, experiencing transient unwrapping transitions that last from 10 to 50 ms every 250 ms (77). In addition, the entry and exit DNA ends of the nucleosome breathe, and the nucleosomes twist and slide on the DNA, as recently observed in all-atom molecular dynamics simulations (5). Histone post-translational modifications further regulate such dynamics, facilitating nucleosome unwrapping and enhancing the subsequent histone release, which makes the DNA more accessible to ETF binding (45).

**Figure 5:**
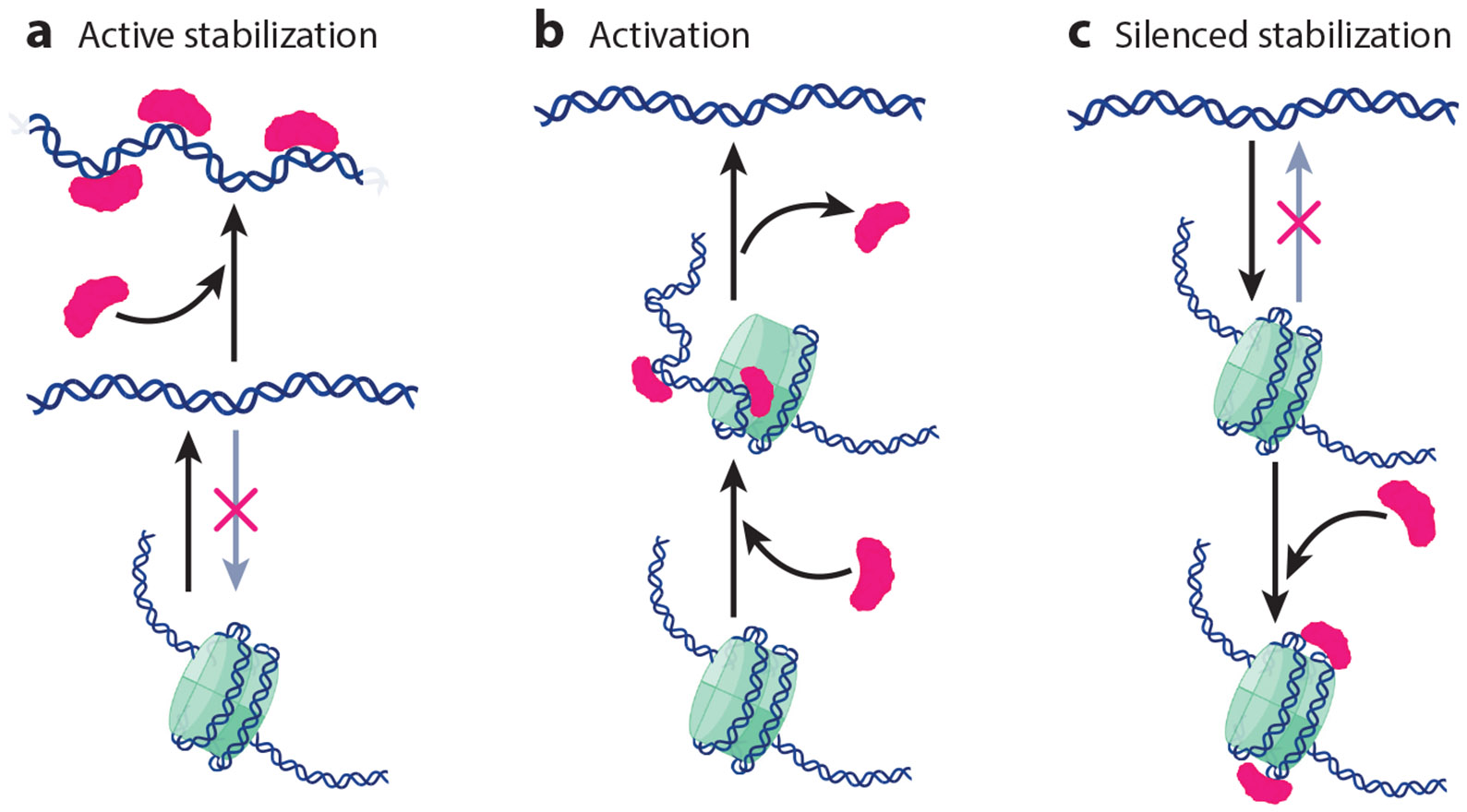
Mechanisms of gene activation and silencing mediated by eukaryotic transcription factor (ETF) interactions with naked DNA and nucleosomes. (*a*) ETF molecules can impede nucleosome wrapping by collectively binding to a naked DNA region, thus stabilizing the active state. (*b*) An ETF that is capable of targeting nucleosomes could induce, or favor, the mechanical unwrapping of the nucleosome DNA. This ultimately results in the activation of a silenced region, with the ETF acting as a pioneer transcription factor. (*c*) An ETF that binds nucleosomes with higher affinity than that with which it binds naked DNA can stabilize a silenced region by collective binding to the nucleosomes.

ETFs must thus work around nucleosomes, either evading, competing with, or even targeting them. All ETFs can obviously access DNA once it is cleared out of nucleosomes. ETFs can also exploit nucleosome dynamics to gain access to otherwise buried DNA segments. One result of such competition appears to be accelerated DNA dissociation rates for ETFs in vivo, up to 1,000-fold for Gal4 (80). In addition, ETFs can actively participate in nucleosome (dis)assembly and hence control the activation and/or silencing of target genomic regions. Three direct functions that ETFs could play in nucleosome (dis)assembly are shown schematically in Figure 5. The first function involves direct binding competition with histones for naked DNA. In this case, the collective action of several factor molecules, whether copies of the same ETF or of several partners, can block nucleosome assembly by dynamically occupying multiple local sites to keep the locus transcriptionally active (Figure 5a). This role does not require direct ETF–nucleosome interactions and has been recently analyzed with the use of theoretical models (41, 96). Here collective action is again important to build up a sufficient free energy input. Enabling such collective actions in regions of interest might be yet another important function for the STR clusters of transcription antennas acting together with a promiscuous recognition code.

In addition, a group of ETFs, called pioneer transcription factors, have the ability to recognize and bind to their sequence motifs within nucleosomes and hence target silenced DNA to start gene expression programs (138). A high-throughput survey found that more than half of 400 tested ETFs were capable of targeting their cognate motifs in nucleosomes, usually with access more limited than that to naked DNA and with various binding poses (140). This study identified two properties that enable nucleosome targeting: (*a*) a recognition motif that is short enough (≤8 bp) to be fully displayable on the nucleosome surface, and (*b*) an open DNA interface that avoids histone clashes (140). ETFs that target nucleosomes can perform two additional nucleosome positioning functions. The second function occurs if ETF binding destabilizes the nucleosome to thus initiate or help chromatin remodelers with nucleosome unwrapping to activate silenced DNA (Figure 5b). Nucleosome destabilization should occur if the ETF binds nucleosomes with lower affinity than that with which it binds naked DNA, such that binding promotes the latter. In this regard, Rap1 binds nucleosomes with dwell times shorter than those with which it binds naked DNA and disrupts chromatin by preventing internucleosome contacts (95). Reb1 targets the DNA at the entry/exit region and promotes a partially unwrapped state of the nucleosome (31). Gal4, which binds to the inner nucleosome region with accessibility 100-fold-lower than that with which it binds to the entry/exit region, seems to slow down nucleosome rewrapping by binding competition (32). The p53 tetramer can also bind nucleosomes when its 20-bp recognition motif is placed at the entry/exit region, thereby pulling away the DNA from the histone core (101). Cryo-electron microscopy structures of pioneer ETFs SOX11, SOX2, and OCT4 bound to nucleosomes support the same scenario, with these factors bound at the entry/exit region while distorting nucleosome wrapping (30, 91). The third function occurs when ETF binding stabilizes the nucleosome, which would consolidate the DNA in silenced form with the ETF in a repressor role (Figure 5c). This function has not been investigated at an equivalent depth. However, in a recent study we found that enHD binds nucleosomes containing multiple cognate sites with significantly higher affinity than that with which it binds to the same DNA in naked form (51). From those results we estimated that the simultaneous binding of three enHD molecules to one nucleosome could stabilize it by about half of the work required to mechanically unravel its first 60–65 bp (51).

## FUTURE DIRECTIONS

The realization that at least some ETFs use a promiscuously tiered recognition code has important practical consequences for understanding the complex occupancy patterns found in vivo, as well as the interplay between ETF and eukaryotic regulatory elements. It will be important to determine how widespread promiscuous recognition is in ETFs and to understand the different degrees of promiscuity that might occur in nature. To that end, new high-throughput methods that shift from selection of the fittest to the detection of midlevel-affinity ranges (110) hold great promise for revisiting ETF recognition logos.

The interplay between ETFs and the STR clusters of eukaryotic regulatory regions takes on entirely new functional meanings once promiscuous recognition is considered. These regions become transcription antennas for ETF colocalization in complex environments. But transcription antennas can play even wider roles by recruiting full transcription hubs containing all the components required for transcription to genomic regions of interest. This recruitment process could occur via individual interactions of each ETF with the antenna or via the interactions of select components of the phase-separated condensates that self-organize using transient interactions between the IDRs of multiple protein partners (15, 20, 23). Exploring these exquisitely sophisticated interplays opens an exciting new avenue of research.

Oddly enough, there still are fundamental gaps in our understanding of protein–DNA interaction kinetics. For instance, we need accurate estimates of the true 3D diffusion-collision rates for more transcription factors, preferably ETFs. This information is critical to better understand the association-dissociation rate trade-offs that define target site affinity and the dynamic response of ETFs. It is also essential to ascertain the exact contributions of electrostatic steering. Additional experimental approaches might ultimately be needed for meeting those purposes. One possibility would be to use single-molecule spectroscopy to resolve the kinetics of binding to short DNA molecules that minimize noncognate binding at subnanomolar concentrations (minimal intersegment crossing) with microsecond resolution. Enhanced single-molecule imaging experiments capable of resolving local binding and exchange kinetics would also be extremely useful to determine the kinetic effects of the surrounding DNA sequence in real time, and ultimately in vivo.

Similar considerations apply to the ETF mechanisms for scanning naked DNA. Current understanding is shaped by studies of enzymes and transcription factors that bind DNA with large interfaces that mechanically support the sliding motion. Our recent work on enHD uncovered a hybrid mechanism that is highly dynamic and relies on electrostatic attraction as the only sustaining force. This hybrid mechanism combines local scanning sweeps with fast long jumps on DNA of approximately 500 bp, which seems perfectly suited for navigating the complex binding landscapes of transcription antennas. A similar hybrid mechanism could be potentially used by other ETFs that bind DNA with an open interface. It would thus be important to investigate whether other ETFs use such hybrid DNA scanning mechanisms. There also are pending experimental issues. Particularly, the quick exchange between sliding, hopping, and gliding modes that likely occurs during local scanning by enHD and other ETFs remains unresolved. Resolving these microscopic processes will require single-molecule techniques with enhanced time and space resolutions (86). The mechanism should also be explored in live cells, but this will require incorporating fast super-resolution into current in vivo single-molecule tracking methods (48, 102).

The mechanisms for conformational control in search and recognition are another focal area of interest for future biophysical studies. Here we find particularly exciting the possibility of conformational control exerted directly by the DBD, with or without help from flanking IDRs. Although the DBD of ETFs form defined 3D structures that are generally maintained in their complexes with DNA (47), a simple sequence analysis predicts that most of the known eukaryotic DBDs are intrinsically disordered (Figure 4b,c). Although this prediction might be overstated, it does indicate that DBDs have inherently flexible conformational ensembles likely to be naturally responsive to the negative electric field of DNA. We thus anticipate that many other ETFs may use a conformational control mechanism like that found on enHD.

Finally, an exciting research area is defined by the interactions between ETFs and nucleosomes. The realization that many ETFs can target DNA wrapped in nucleosomes opens a realm of unforeseen molecular functionalities and mechanisms. By targeting DNA on nucleosomes, ETFs can actively participate in the dynamic control of chromatinization, whether as pioneers that start full gene expression programs (138) or that maintain them silenced. These new functionalities are just beginning to be investigated at the biophysical level; thus, we anticipate seeing important advances in the near future.
